# Measurement properties of the Regular Physical Exercise Adherence Scale (REPEAS) in individuals with chronic pain

**DOI:** 10.1186/s12889-024-19297-w

**Published:** 2024-07-03

**Authors:** Cezar Augusto Brito Pinheiro, Daniela Bassi-Dibai, André Pontes-Silva, Fábio Henrique Ferreira Pereira, Jocassia Silva Pinheiro, Cid André Fidelis-de-Paula-Gomes, Almir Vieira Dibai-Filho

**Affiliations:** 1 Postgraduate Program in Adult Health, Universidade Federal do Maranhão, São Luís, MA Brazil; 2 Postgraduate Program in Environment, Universidade Ceuma, São Luís, MA Brazil; 3 Postgraduate Program in Physical Therapy, Department of Physical Therapy, Universidade Federal de São Carlos, São Carlos, SP Brazil; 4 Postgraduate Program in Rehabilitation and Functional Performance, Universidade de São Paulo, Ribeirão Preto, SP Brazil; 5 Postgraduate Program in Rehabilitation Science, Universidade Nove de Julho, São Paulo, SP Brazil

**Keywords:** Chonic pain, Exercise, Sedentary behavior, Public health

## Abstract

**Objective:**

To examine the measurement properties of the Regular Physical Exercise Adherence Scale (REPEAS) in Brazilians with chronic pain.

**Methods:**

Cross-sectional and longitudinal design (washout period for reliability). The study was conducted in two Brazilian states, Maranhão and São Paulo, and included Brazilian adults, irregular exercisers, former exercisers or non-exercise practitioners, aged 18 to 59 years and with chronic pain. The instruments used in this study were: the REPEAS, the Numerical Pain Rating Scale (NPRS), the Baecke Habitual Physical Activity Questionnaire (BHPAQ), the Pain Self-Efficacy Questionnaire (PSEQ), and the Roland-Morris Disability Questionnaire for general pain (RMDQ-g). The evaluation focused on structural validity, construct validity, reliability (with standard error of measurement and minimum detectable change), internal consistency, and floor and ceiling effects.

**Results:**

The two-dimensional structure was tested through confirmatory factor analysis, which resulted in adequate fit indeces: chi-square values/degrees of freedom = 1.541, Tucker-Lewis Index = 0.966, comparative fit index = 0.974, root mean square error of approximation = 0.074, and standardized root mean square residual = 0.068. Additionally, satisfactory factor loadings (> 0.40) were obtained. Test-retest reliability and internal consistency were adequate for the environmental factors domain (intraclass correlation coefficient [ICC] = 0.79, Cronbach’s alpha = 0.88) and the personal factors domain (ICC = 0.97, Cronbach’s alpha = 0.93). In hypothesis testing for construct validity, we observed a significant correlation with magnitude below 0.30 of the environmental factors domain of the REPEAS with RMDQ-g, PSEQ and sport domain of the BHPAQ. For the personal factors domain, we observed a significant correlation with a magnitude of 0.30 to 0.50 with RMDQ-g, PSEQ, and sport domain of the BHPAQ, and below 0.30 with leisure domain of the BHPAQ. No floor or ceiling effects were found for the REPEAS domains.

**Conclusion:**

The REPEAS is a valid instrument with a two-dimensional internal structure consisting of 12 items. It has a reliable construct and is suitable for use in the clinical and epidemiological context for adults with chronic pain in Brazil.

## Introduction

Chronic pain is pain that persists beyond the normal tissue healing time (typically 12 weeks) [1]. It contributes to disability, anxiety, depression, sleep disturbance, poor quality of life, and overall health care costs [2]. For many years, the treatment of choice for chronic pain included recommendations for rest and inactivity [3]. However, we currently know that physical exercise has specific benefits in reducing the severity of chronic pain, as well as more general benefits associated with increasing quality of life [1–3].

Physical exercise is defined as a type of physical activity that is planned, structured, and repetitive, with the goal of improving or maintaining physical fitness levels [4, 5]. Therefore, while all physical exercise is a form of physical activity, not all physical activity is considered physical exercise [4, 5]. Physical inactivity is a significant modifiable health risk behavior and ranks as the fourth leading risk factor for mortality [4, 5].

Recent studies suggest that physical activity levels have decreased significantly in countries with higher per capita income, as well as in middle and low-income countries [6, 7]. Sedentary behavior is defined as activities that do not significantly increase energy expenditure above the resting level [8]. In many countries, adults do not adhere to the recommended levels of aerobic exercise and muscle strengthening as proposed by the World Health Organization (WHO) [4, 5].

In complement, the exercise adherence is largely impacted by sociodemographic factors and lifestyle of the population [9]. Environmental and safety factors are also found to impact adherence [10]. For individuals with chronic pain, the literature consistently supports exercise as one of the most effective therapeutic strategies, regardless of the type of exercise modality [1]. Chronic pain is typically described as diffuse pain lasting for more than three months. Chronic pain is primarily associated with the nociplastic category of pain mechanisms, where nociception is altered due to probable neuronal dysregulation, without any apparent tissue damage [11].

Given the importance of regular exercise for people with chronic pain, it is crucial to identify the barriers to proper adherence to this therapeutic and health-promoting modality. Regular exercise promotes improvement in pain sensitization through the release of endogenous opioids and beta-endorphins that act on hypoalgesia [12] and improves physical function by reducing pain [13].

Currently, the scientific literature only provides validation for the Exercise Adherence Rating Scale (EARS) in Brazil [14]. This tool assesses adherence to exercise prescribed by healthcare professionals for individuals with chronic low back pain. However, while the EARS presents adequate measurement properties, it lacks items related to environmental factors and has characteristics that are more focused on clinical monitoring of patients rather than addressing the context of the epidemiological profile. Additionally, it was validated for a specific population with chronic pain, specifically low back pain [14].

Therefore, in this scenario, the Regular Physical Exercise Adherence Scale (REPEAS) was created and validated in 2023 for the general population. It consists of items that investigate the barriers to adherence to regular exercise, distributed in two domains: environmental factors and personal factors [15]. As such, REPEAS fills an important gap in the management of patients with chronic pain. As such, we aimed to examine the measurement properties (structural validity, reliability, internal consistency, and construct validity) of the REPEAS in Brazilians with chronic pain.

## Methods

### Study design

This is a validation study for a questionnaire with a cross-sectional and longitudinal design (washout period for reliability), conducted in accordance with the COnsensus-based Standards for the selection of health Measurement INstruments (COSMIN) guidelines [16, 17]. Data was collected in two different states in Brazil: Maranhão (northeast) and São Paulo (southeast). The project received approval from the research ethics committee of the institution (report number: 5.328.899).

### Sampling

The COSMIN recommendation was utilized, which considers 7 times the number of items in the instrument as an adequate sample size, provided that the minimum sample size is 100 participants [16, 17]. The sample consisted of adult individuals of both sexes, aged between 18 and 59 years, who self-reported chronic pain (for more than 3 months) in any region of the body, and included irregular exercisers, former exercisers, and non-exercise practitioners.

Individuals were recruited through social network advertising and in-person by the research team. Non-inclusion criteria included individuals who were not native to Brazil, had medical contraindications to performing physical exercises, or had a medical diagnosis of severe cognitive or psychiatric changes.

### Eligibility criteria

This study enrolled patients with pain ≥ 3 points on the Numerical Pain Rating Scale (NPRS) [18] at the time of assessment [19] and with pain persisting for at least 3 months at a level similar to that at the time of assessment [2, 20]. Eligible patients were literate in Brazilian Portuguese, had no diagnosed cognitive dysfunction, and were at least 18 years of age.

### Assessement

Data was collected online using the Google Forms platform (Mountain View, CA, USA). The online form included items related to sociodemographic data, personal and clinical characteristics. Subsequently, the NPRS [18], REPEAS [15], Baecke Habitual Physical Activity Questionnaire (BHPAQ) [21], Pain Self-Efficacy Questionnaire (PSEQ) [22], and Roland-Morris Disability Questionnaire for general pain (RMDQ-g) were presented [23].

### Numerical pain rating scale (NPRS)

NPRS is a scale used to quantify the pain intensity using a sequence of 11 numbers, in which 0 represents “no pain” and 10 “the worst pain imaginable”. The pain intensity was assessed at rest and after active spinal movements. This scale is validated for Portuguese [18]. This scale was used to characterize the sample.

### Regular physical exercise adherence scale (REPEAS)

The focus of this study is on REPEAS, a tool used to evaluate the factors that affect adherence to physical exercise. The tool consists of a list of physical, emotional, and environmental situations that can either facilitate or hinder regular physical exercise. There are 12 items distributed in two domains: environmental factors domain (items 1 to 5) and personal factors domain (items 6 to 12). The respondent must indicate on a scale from 0 to 10 the answer option that best indicates these situations, in which 0 means “Does not make it difficult to practice physical exercise” and 10 means “It makes it very difficult to practice physical exercise”. To calculate the score per domain, add up the values of the responses given to each item and divide by the number of items answered. This will generate a score ranging from 0 to 10, which should then be multiplied by 10 to obtain a score from 0 to 100. A higher score indicates poorer adherence to physical exercise [15].

### Baecke habitual physical activity questionnaire (BHPAQ)

The BHPAQ is a self-administered tool that assesses physical activity over the past 12 months through self-report. It comprises 16 items, categorized into three domains: occupational (items 1–8), sport (items 9–12), and leisure (items 13–16). To calculate the final score, each domain must be considered separately. The total score ranges from 1 to 5, with higher scores indicating greater habitual physical activity. The BHPAQ has been adapted and validated for Brazilian Portuguese [21].

### Pain self-efficacy questionnaire (PSEQ)

The PSEQ is a validated instrument for the Brazilian population. It consists of 10 items, each scored on a Likert scale from 0 to 6, with 0 indicating ‘not at all confident’ and 6 indicating ‘completely confident’. The total score ranges from 0 to 60, with higher scores indicating greater self-efficacy [22].

### Roland-Morris disability questionnaire for general pain (RMDQ-g)

The RMDQ-g is a valid instrument for the Brazilian population. It consists of 24 items, each scored as either 0 (no) or 1 (yes). The total score ranges from 0 to 24, with higher scores indicating greater disability [23].

### Statistical analysis

SPSS software version 17.0 (Chicago, IL, USA) was used to process data on reliability (with standard error of measurement and minimum detectable change), internal consistency, construct validity, and other descriptive variables. The presentation of these variables is in the form of mean and standard deviation or absolute number and percentage. Structural validity was assessed using confirmatory factor analysis (CFA) with the software R Studio (Boston, MA, USA) and the lavaan and semPlot packages [24].

The REPEAS was scored on a Likert scale (ordinal data). Thus, the researchers performed CFA by implementing a polychoric matrix and the robust diagonally weighted least squares (RDWLS) extraction method. We used the following fit indices: root mean square error of approximation (RMSEA) with a 90% confidence interval (CI), comparative fit index (CFI), Tucker-Lewis index (TLI), standardized root mean square residual (SRMR), and chi-square/degrees of freedom (DF).

The study considered values greater than 0.90 as adequate for CFI and TLI, and values less than 0.08 as adequate for RMSEA and SRMR. Values below 3.00 were considered adequate for the interpretation of chi-square/DF [25, 26]. Factor loadings equal to or greater than 0.40 were considered adequate for the domain [24].

Reliability (with standard error of measurement and minimum detectable change) was assessed using a test-retest model. A subsample of 50 participants answered the REPEAS twice, with a 7-day washout period between responses. The intraclass correlation coefficient (ICC) was used to determine reliability, with a cutoff point of acceptability set at a value greater than 0.75 [27]. Additionally, we calculated the standard error of measurement and minimum detectable change [19]. The internal consistency of each domain was calculated using Cronbach’s alpha, with appropriate values considered to be between 0.70 and 0.95 [28].

In the hypothesis testing for construct validity, we conducted the Kolmogorov-Smirnov normality test and used Spearman’s correlation coefficient (rho) to correlate the REPEAS with the BHPAQ, PSEQ, and RMDQ-g (due to non-normality of data distribution). The significance level was set at 0.05. Based on a previous study [14] and the nature of the constructs assessed by the instruments of the present study, we define the following hypotheses listed below.

- for the personal factors domain: significant and negative correlation (magnitude less than 0.30) with the PSEQ and RMDQ-g; significant and negative correlation (correlation magnitude between 0.30 and 0.50) with the sport domain of the BHPAQ; significant and negative correlation (magnitude less than 0.30) with the occupational and leisure domains of the BHPAQ;
- for the environmental factors domain: significant and negative correlation (correlation magnitude less than 0.30) with PSEQ, RMDQ-g and the BHPAQ domains.

The present study evaluated ceiling and floor effects. These effects occur when more than 15% of study participants reach the minimum or maximum values for the total questionnaire score, as defined [29].

## Results

### Sample characterization

The sample consists of 100 individuals, with the majority being female (72%). They are young adults, approximately 33 years old, and are married (49%). As shown in Table 1, most have completed higher education or more years of study. In terms of reported pain locations, the majority of participants reported low back pain (57%), followed by shoulder pain (26%) and neck pain (19%). Details on the pain locations are in Table 2 and descriptive values of the used questionnaires are in Table 3.

**Table 1:** Personal and social variables (*n* = 100)

Variables	% or mean (standard-deviation)	
Age (years)	33.3 (11.0)	
Sex (female)	72%	
Body mass (kg)	73.8 (18.32)	
Stature (cm)	166.3 (8.45)	
Body mass index (kg/m²)	26.7 (6.25)	
Marital status		
married	49%	
divorced	3%	
single	47%	
widower	1%	
Education		
incomplete primary education	1%	
high school completed	15%	
incomplete high school	1%	
completed higher education	25%	
incomplete tertiary education	18%	
completed postgraduate	25%	
incomplete postgraduate	15%	
Physical exercise		
former practitioner	42%	
never practiced	6%	
irregular practitioner	52%	
Smoker		
ex-smoker	6%	
no	88%	
yes	6%	
Pain time (months)	44.26 (40.38)	

**Table 2:** Pain locations reported by study participants (*n* = 100)

Pain locations	%
Lumbar spine	57
Shoulder	26
Cervical spine	19
Knee	17
Thoracic spine	14
Leg	14
Head	12
Feet	10
Wrist	8
Hand	6
Hip	2
Arm	2
Elbow	1
Face	1
Other	14

**Table 3:** Descriptive values of the used questionnaires (*n* = 100)

Questinnaire	Mean	Standard-deviation
RMDQ-g (score, 0–24)	17.12	5.63
PSEQ (score, 0–60)	45.84	13.36
BHPAQ		
occupational (score, 1–5)	2.65	0.62
sport (score, 1–5)	2.13	0.91
leisure (score, 1–5)	2.41	0.66
REPEAS		
environmental factors (score, 0-100)	51.54	26.63
personal factors (score, 0-100)	44.12	24.01
NPRS (score, 0–10)	4.96	1.98

RMDQ-g: Roland-Morris Disability Questionnaire for general pain; PSEQ: Pain Self-Efficacy Questionnaire; BHPAQ: Baecke Habitual Physical Activity Questionnaire; REPEAS: Regular Physical Exercise Adherence Scale; NPRS: Numeric Pain Rating Scale

### Structural validity

**Fig. 1:**
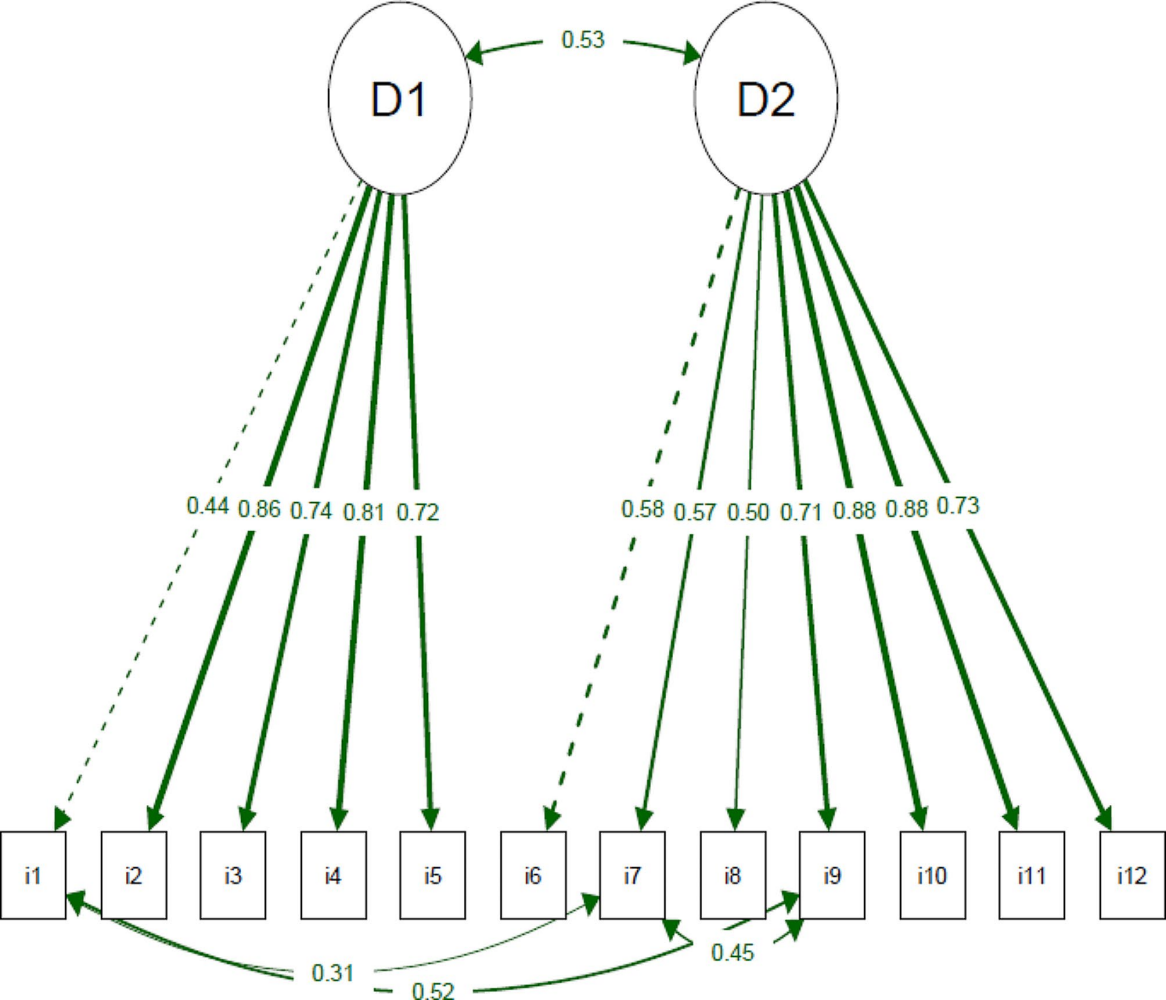
The path diagram of the Regular Physical Exercise Adherence Scale (REPEAS) displays factor loadings that satisfactorily indicate the relationship between domains and items (> 0.40). The dashed line represents the first item in the domain. The thickness of the line corresponds to the factor loading. D1: Environmental factors; D2: Personal factors

The two-dimensional structure of the REPEAS was tested using CFA on items 1 to 5 in the environmental factors domain and 6 to 12 in the personal factors domain. Adequate fit indices were observed: chi-square/DF = 1.541, CFI = 0.974, TLI = 0.966, RMSEA = 0.074 (90% CI = 0.038 to 0.105), and SRMR = 0.068. Additionally, the factor loadings of the domains explaining the scale items were satisfactory (> 0.40), as shown in Fig. 1.

### Reliability and internal consistency

Table 4 presents the values for test-retest reliability (with standard error of measurement and minimum detectable change) and internal consistency. The ICC was 0.79 and Cronbach’s alpha was 0.88 for environmental factors, while the ICC was 0.87 and Cronbach’s alpha was 0.93 for personal factors.

**Table 4:** Test-retest reliability and internal consistency of the regular physical exercise adherence scale (*n* = 50)

Values	Domains	
	Environmental factors	Personal factors
Test: mean (standard-deviation)	41.70 (26.54)	38.62 (23.49)
Retest: mean (standard-deviation)	47.02 (26.37)	32.02 (24.33)
Intraclass correlation coefficient	0.79	0.87
Standard error of measurement	11.95	8.60
Minimum detectable change	33.12	23.84
Cronbach’s alpha	0.88	0.93

### Hypothesis testing for construct validity

We confirmed 70% of the hypotheses defined a priori, as shown in Table 5. For the environmental factors domain, we observed a significant correlation with a magnitude below 0.30 with RMDQ-g, PSEQ and sport domain of the BHPAQ. For the personal factors domain, we observed a significant correlation with a magnitude of 0.30 to 0.50 with RMDQ-g, PSEQ, and sport domain of the BHPAQ, and below 0.30 with leisure domain of the BHPAQ.

**Table 5:** Construct validity of domains of the regular physical exercise adherence scale (*n* = 100)

Variáveis	Environmental factors		Personal factors	
	rho	*p*	rho	*p*
RMDQ-g	-0.149	0.041*	-0.333	0.001*
PSEQ	-0.278	0.005*	-0.332	0.001*
BHPAQ				
occupational	0.194	0.054	0.038	0.705
sport	-0.211	0.035*	-0.372	< 0.001*
leisure	0.004	0.972	-0.222	0.027*

RMDQ-g: Roland-Morris Disability Questionnaire for general pain; PSEQ: Pain Self-Efficacy Questionnaire; BHPAQ: Baecke Habitual Physical Activity Questionnaire.

### Floor and ceiling effects

In the domain of environmental factors, none of the participants achieved the maximum score of 100 points, while 5% of the sample scored the minimum of 0. In the personal factors domain, no participant scored either the minimum or maximum. Thus, no floor or ceiling effects were identified in the REPEAS domains.

## Discussion

The objective of this study was to facilitate the use of the newly developed REPEAS instrument for individuals in Brazil who suffer from chronic pain. The REPEAS has a two-dimensional structure comprising of 12 items and has demonstrated satisfactory structural validity, construct validity, reliability, and internal consistency.

When examining the measurement properties of the original REPEAS instrument in healthy adults, the researchers reported fit indices for the instrument’s structural validity that were slightly better than those found in the present study (CFI = 0.973, TLI = 0.966, RMSEA = 0.075, and SRMR = 0.062) [15]. However, both structures within the model’s acceptability cutoff points. Therefore, our study also confirms the two-dimensional structure of the instrument.

The researchers reported adequate reliability values for the original version of REPEAS [15]. Specifically, they found an ICC of 0.86 and 0.94 for the environmental factors and personal domains, respectively, as well as a Cronbach’s alpha of 0.90 and 0.91 for the environmental factors and personal domains, respectively. Our study discovered lower reliability values for the environmental and personal domains, with ICC values of 0.79 and 0.87, respectively, and Cronbach’s alpha values of 0.88 and 0.93, respectively. However, all values are above the acceptable cutoff points of ICC > 0.75 and Cronbach’s alpha > 0.70.

The validation of the original version of REPEAS involved a comparison between different groups, including regular practitioners and ex-practitioners/non-practitioners. In our study, we correlated measures known for patients with pain and found a significant correlation between the REPEAS domains and self-efficacy, physical activity related to sports and leisure, functional disability (correlation magnitude ranging from − 0.149 to -0.372).

The EARS investigates adherence to exercise prescribed at home by health professionals for people with chronic low back pain. Newman-Beinart et al. [30] developed the EARS, and the Brazilian version of the instrument demonstrated adequate reliability (ICC of 0.91) and internal consistency (Cronbach’s alpha of 0.88). Additionally, the EARS construct was deemed adequate, exhibiting correlation magnitudes comparable to those found in the present study. The correlations ranged from − 0.22 to -0.37 with constructs such as anxiety, depression, catastrophizing, avoidance, and disability. The only exception was pain intensity, which showed a higher correlation magnitude (rho = -0.52) [14].

In addition, the Adherence to Exercise for Musculoskeletal Pain Tool (ATEMPT) was recently created in English for individuals with chronic musculoskeletal pain. The authors reported adequate reliability (ICC of 0.78 and 0.88 for the 6-item version) and internal consistency (Cronbach’s alpha of 0.83 and 0.88 for the 6-item version) [31]. However, construct validity was not assessed.

The REPEAS has several positive attributes, including appropriate domains based on factor analysis, a broad scope that is not limited to a specific pain group, a concise 12-item format that is easy to complete, and its use in both clinical and epidemiological contexts. Additionally, there are no costs or fees associated with using the REPEAS. In terms of practical implications, the REPEAS is the first instrument adapted for people with chronic pain and will be useful for investigating barriers to adherence to regular exercise, whether due to environmental or personal factors. Furthermore, it is an instrument with few items, taking into account international recommendations for instruments that are easy to use in the clinical routine of evaluating patients with chronic pain.

Our study has limitations that must be considered. The REPEAS tool only assesses environmental and personal barriers to regular physical exercise in people with chronic pain. It does not assess other barriers that health professionals may encounter in their practice. The results of this study are only applicable to the Brazilian population. Therefore, it is necessary to translate, adapt and validate the tool for use in other countries and cultures. Finally, we suggest additional studies to analyze whether the results of personal and environmental factors influence exercise adherence in this population and what the clinical implications are.

## Conclusion

The REPEAS is a valid instrument with a two-dimensional internal structure consisting of 12 items. It has a reliable construct and is suitable for use in the clinical and epidemiological context for adults with chronic pain in Brazil.

## Data Availability

The data and materials in this paper are available from the corresponding author on request (André Pontes-Silva).

## Abbreviations

CFA: Confrmatory Factor Analysis
CFI: Comparative fit index
COSMIN: COnsensusbased Standards for the selection of health Measurement INstruments
DF: Degree of freedom
NPRS: 11-Item Numeric Pain Rating Scale
RDWLS: Robust Diagonally Weighted Least Squares
RHO: Spearman’s Correlation Coefficient
RMSEA: Root Mean Square Error of Approximation
REPEAS: Regular Physical Exercise Adherence Scale
BHPAQ: Baecke Habitual Physical Activity Questionnaire
PSEQ: Pain Self-Efficacy Questionnaire
RMDQ-g: Roland-Morris Disability Questionnaire for general pain
WHO: World Health Organization
